# KY-MRSA: a comprehensive review of methicillin-resistant *Staphylococcus aureus* polymerase chain reaction nasal screening practices across nine institutions in Kentucky

**DOI:** 10.1017/ash.2025.10201

**Published:** 2025-10-30

**Authors:** Clover N. Truong, Wes M. Johnson, Jamison Montes de Oca, Sarah E. Moore, Matthew Song, Elena A. Swingler, Ashley M. Wilde

**Affiliations:** Department of Pharmacy Services, Norton Healthcare, Louisville, KY, USA

## Abstract

**Objective:**

To describe the screening practice of nasal methicillin-resistant *Staphylococcus aureus* (MRSA) polymerase chain reaction (PCR) among nine health organizations in Kentucky.

**Methods:**

The Kentucky Antimicrobial Stewardship Innovation Consortium (KASIC) invited its Advisory Board members to share their nasal MRSA PCR protocols and guidelines. The documents were examined to highlight institutional similarities and differences.

**Results:**

Nine health systems, including both community hospitals and academic medical centers, responded to the KASIC request. Most systems with nasal MRSA PCR testing capacity had established protocols or guidelines to support its appropriate use. All institutions recommended nasal MRSA PCR for pneumonia indications while three organizations also used it for non-pneumonia indications. None of these institutions permitted pharmacists to discontinue anti-MRSA antibiotics per protocol.

**Conclusions:**

This study provides the first statewide overview of nasal MRSA PCR screening practices, offering stewardship programs a framework to customize their own protocols and guidelines.

## Introduction

The Centers for Disease Control and Prevention considers methicillin-resistant *Staphylococcus aureus* (MRSA) as a serious threat because it can cause difficult-to-treat infections with high mortality.^1^ In Kentucky, MRSA rates remain >40% among *S. aureus* isolates.^2^ Current community-acquired pneumonia (CAP) guideline recommends empiric MRSA coverage in patients with risk factors (prior respiratory isolation or recent hospitalization receiving parenteral antibiotics).^3^ Hospital-acquired (HAP)/ventilator-associated pneumonia (VAP) guideline recommends anti-MRSA antimicrobial in patients treated in units where >10–20% of *S. aureus* isolates are methicillin resistant which essentially means all HAP/VAP patients receive empiric anti-MRSA in practice.^4^ Anti-MRSA activity is also recommended empirically in purulent skin soft tissue infection, bone and joint infection, central nervous system (CNS) infection, and surgical prophylaxis in patients colonized with MRSA.^5,6,7,8^ While this empirical approach helps cover for potential invasive MRSA infection, prolonged MRSA coverage can lead to unnecessary exposure, higher risk for developing resistance such as vancomycin-resistant *Enterococcus*, adverse reactions, longer hospital stays, and higher treatment costs.^9^ Therefore, finding reliable screening tools to safely discontinue anti-MRSA therapy is essential in antimicrobial stewardship programs.

*S. aureus* often colonizes in the human nares, and its colonization frequently precedes the onset of clinical infection.^9^ There are two types of nasal MRSA swabs used in clinical practice to detect MRSA colonization: culture and polymerase chain reaction (PCR).^9^ The advantage of nasal MRSA PCR is its faster turnaround time and its higher sensitivity compared to nasal MRSA culture, but comes at an increased cost.^9^ Recent literature has shown that nasal MRSA PCR can be utilized as an antimicrobial stewardship tool to de-escalate anti-MRSA therapy in pneumonia, with limited studies suggesting its role for non-pneumonia indications.^9^ Although some institutions have shared their nasal MRSA PCR protocol and experience, no study has provided a comprehensive review of nasal MRSA PCR screening practice across multiple sites.^10,11^ This gap in information creates challenges for new stewardship programs developing practical nasal MRSA PCR protocols/guidelines as staffing and clinical practice models vary between different types of institutions (academic medical centers vs community hospitals; large health-system vs small hospitals). This study aims to summarize and emphasize the similarities and differences in nasal MRSA PCR practices among various institutions in Kentucky.

## Methods

The Kentucky Antimicrobial Stewardship Innovation Consortium (KASIC) was established in 2022 with a goal to promote optimal antimicrobial use across the state. KASIC includes an Advisory Board, consisting of 26 pharmacists who practice in established antimicrobial stewardship programs, and a network of antimicrobial stewardship leads at all adult hospitals and long-term care facilities throughout Kentucky. KASIC asked the Advisory Board members to share their nasal MRSA PCR protocols and guidelines. Protocol was defined as a policy allowing pharmacists to order a laboratory test if a patient meets predetermined criteria without an order from a provider. Guideline was generally considered a recommendation but did not grant pharmacist autonomy to place an order. These documents were subsequently evaluated and analyzed to identify the similarities and differences in how various institutions in Kentucky use nasal MRSA PCRs.

## Results

Nine of ten institutions (90%) represented by the Advisory Board responded to KASIC’s request to share their nasal MRSA PCR practice. Among responding institutions, 7/9 (77.8%) were adult community health-systems with licensed beds ranged from approximately 200 to 2 300, 1/9 (11.1%) was an adult academic health-system with >1,300 beds, and 1/9 (11.1%) was an academic health-system comprised of adult hospitals with total beds >1,000 and a 205-bed pediatric hospital.

**Figure 1.**
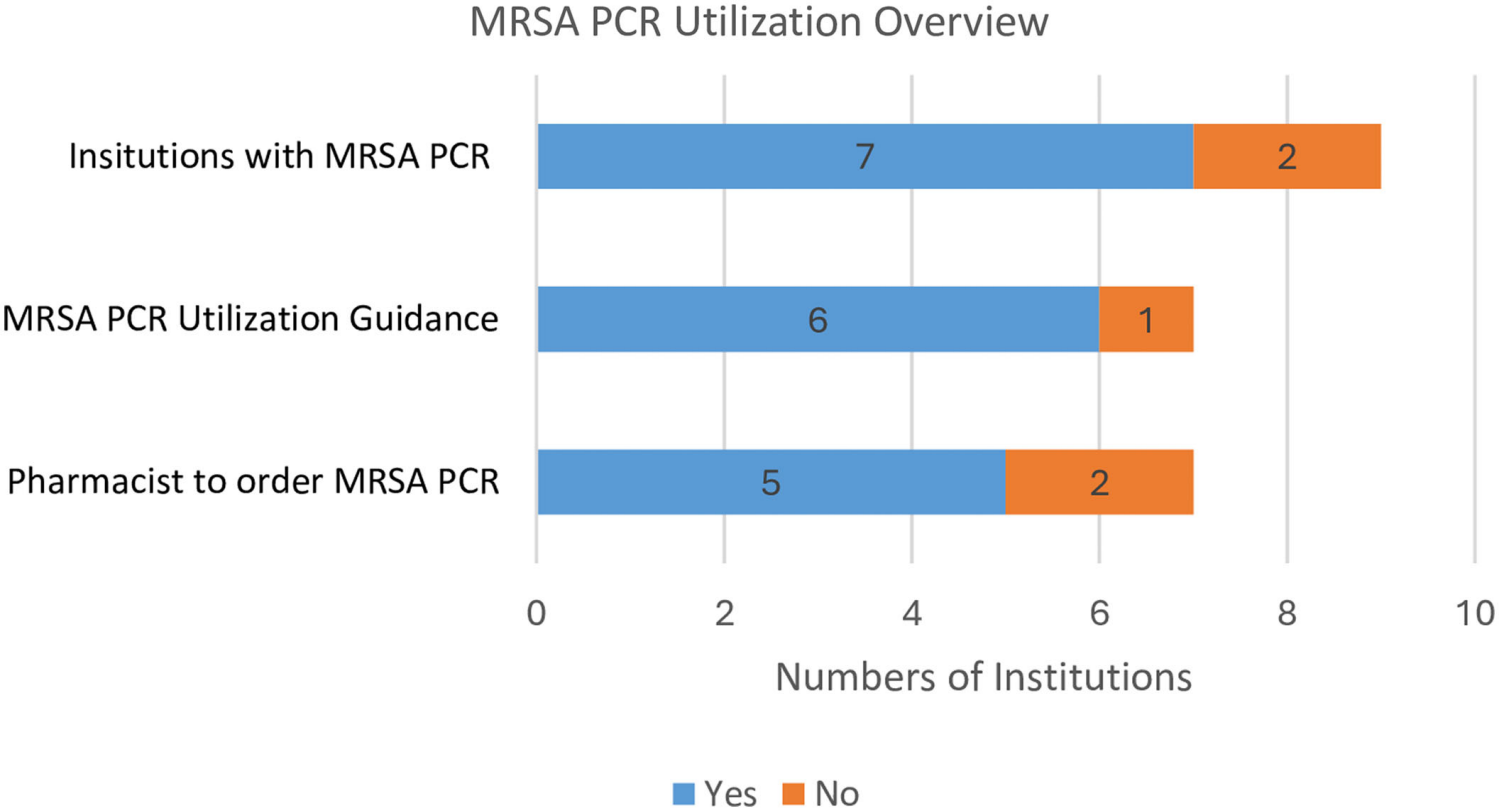
Number of institutions with MRSA PCR utilization.

Among the nine respondents, 7/9 reported having nasal MRSA PCR available (Figure 1). Most institutions employing nasal MRSA PCR swabs established either protocols (5/7; 71.4%) or guideline (1/7; 14.3%) to ensure appropriate use. One institution, without an official policy, noted that their pulmonologist used nasal MRSA PCR screen as needed. All six institutions with established guidelines/protocols endorsed the use of nasal MRSA PCR for pneumonia, with 3/6 (50%) used it for non-pneumonia indications in addition to pneumonia. Among the five institutions with nasal MRSA PCR protocols, 3/5 (60%) allowed pharmacists to order a nasal MRSA PCR upon initiation of IV vancomycin and/or linezolid for pneumonia. 1/5 (20%) enabled pharmacists to order a MRSA PCR no later than 48 hours after MRSA therapy initiated for all indications except CNS, and 1/5 (20%) endorsed pharmacists to order a nasal MRSA PCR within 72 hours of anti-MRSA therapy initiation for certain indications (pneumonia, bone and joint, skin soft tissue infection, and intra-abdominal infection) provided no presence of abscess. No institutions permitted pharmacists to discontinue anti-MRSA therapy per protocol based on a negative nasal MRSA PCR result. There was one healthcare system that established a nasal MRSA PCR guideline for both adult and pediatric patients. In adults, a nasal MRSA PCR was recommended for HAP/VAP. For pediatric patients, this institution suggested using nasal MRSA PCR for complicated CAP (parapneumonic effusion or empyema), preseptal and orbital cellulitis.

### Diagnostic stewardship practice

Inquiries were made regarding diagnostic stewardship efforts of MRSA PCRs. Several institutions provided guidance for nasal MRSA PCR screen based on a patient’s recent MRSA colonization/de-colonization status. Two institutions excluded patients from pharmacist nasal MRSA PCR protocols if patients have existing nasal MRSA PCR result within last 7 days and/or positive MRSA cultures within last 7–14 days. Another healthcare system recommended utilizing available nasal MRSA screen results if obtained within a week prior to pneumonia diagnosis and advised against using nasal MRSA PCR screen in patients having nasal MRSA de-colonization within last 30 days. The abbreviated protocols/guidelines can be found in the **Appendix.**

## Discussion

This review identifies that the majority of institutions employing nasal MRSA PCR screens had established protocols allowing pharmacists to order the test without an order from a provider. Nasal MRSA PCR screens are used universally by these institutions to guide pneumonia therapy. Compelling evidence supporting the use of nasal MRSA PCR in pneumonia drives its widespread adoption in Kentucky hospitals’ policies. The performance of nasal MRSA swabs to predict MRSA pneumonia was reviewed in a meta-analysis of 22 studies (11 with nasal MRSA PCR, 4 utilized nasal MRSA cultures, one had both PCR and cultures, and 6 did not specify MRSA screen method) reported sensitivity of 70.9%, specificity of 90.3%, positive predictive value (PPV) of 44.8%, and negative predictive value (NPV) of 96.5% for all types of pneumonia.^12^ In this analysis, nasal MRSA culture and PCR swabs had higher sensitivities in CAP compared to VAP (85% vs 40.3%).^12^ PPV of nasal MRSA swab remained low regardless the type of pneumonia but has high NPV for both CAP and VAP (98.1% and 94.8% respectively).^12^ Due to low PPV and high NPV of nasal MRSA swabs (culture and PCR) across different types of pneumonia, negative nasal MRSA PCR results prove useful for de-escalating therapy in pneumonia but less helpful when the result is positive. Although KASIC did not focus on impact of MRSA PCR protocols at their institutions, as it was outside the scope of this manuscript, other stewardship programs have reported a reduction in vancomycin duration after implementing pharmacist-led MRSA PCR nasal screening protocols ^10,11^ Additionally, in a retrospective cohort study at Stanford HealthCare, no difference in clinical outcomes including hospital length of stays (LOS), ICU LOS, and in-hospital mortality was found despite shortened vancomycin duration in post MRSA PCR protocol period.^10^ Similar findings were observed in a prospective study at Barnes Jewish Hospital, which used nasal MRSA culture to de-escalate empiric anti-MRSA therapy for critically ill patients with suspected pneumonia.^13^

Despite extensive evidence supporting the use of MRSA PCR for pneumonia, the evidence for nasal MRSA PCR in patients with complicated lung infections (lung empyema or lung abscess) remains limited. A retrospective cohort study at St’s Mary Medical Center in Wyoming evaluated the performance of nasal MRSA PCR in 93 patients with lung empyema and 46 patients with lung abscess.^14^ This study found 80% sensitivity, 84% specificity, 42% PPV, and 97% NPV for empyema and 0% sensitivity, 90% specificity, 0% PPV, and 90% NPV for lung abscess.^14^ The use of nasal MRSA PCR to rule out clinical MRSA infection in adult patients with complicated lung infections warrants caution.

A limited institutions in Kentucky adopted MRSA PCR for non-pneumonia indications, partly due to limited data.^9^ Among non-pneumonia indications, the data is most consistent for intra-abdominal infection with a few retrospective cohort studies reported NPV of 97%.^9^ The performance of MRSA PCR for SSTI is more heterogeneous depending on its location (non-extremity vs extremity), presence of ulcers, and presence of prosthetic implant.^9^ There are no independent studies assessing performance of nasal MRSA PCR in urinary tract infection (UTI); however, a subgroup analysis of patients with urinary cultures at Veterans Affairs inpatient facilities showed 99.2% NPV.^9,15^ It should be noted that MRSA is extremely uncommon cause for UTI, and thus the use of nasal MRSA PCR is likely of low benefit.^9^ No studies have investigated the performance of nasal MRSA PCR in CNS infections.^9^ Given high mortality risk of CNS infections and the use of anti-MRSA agent to target other non-MRSA pathogens (such as drug-resistant *Streptococcus pneumoniae*), the use of nasal MRSA PCR cannot be recommended at this time.^9^ This also aligns with practice in Kentucky, as no institutions endorsed nasal MRSA PCR screen to guide therapy for CNS indications.

An important aspect of MRSA PCR protocol/guidance is determining the utility of nasal MRSA PCR in patients receiving anti-MRSA therapies or MRSA de-colonization. Only two institutions in Kentucky limited the ordering of nasal MRSA PCR to within 48–72 hours of initiation of anti-MRSA antibiotics whereas another institution suggested avoiding using nasal MRSA PCR screen in patients undergoing de-colonization within last 30 days. This was in part due to scarce data examining durability of nasal MRSA PCR positivity for patients receiving anti-MRSA antibiotics or undergoing de-colonization. A study at Upstate University Hospital demonstrated that a positive nasal MRSA PCR typically persisted after 48–96 hours of continuous systemic anti-MRSA antibiotics.^16^ Additionally, a retrospective observational cohort study showed reduced NPV of nasal MRSA PCR screen for pulmonary infections after mupirocin administration.^17^ Decisions to order nasal MRSA PCR screen and de-escalate based on negative result in patients receiving anti-MRSA therapies and/or MRSA de-colonization should be individualized based on exposure duration of anti-MRSA antibiotic and/or topical decolonization (ie, negative nasal MRSA PCR in patients received less than 48 h of anti-MRSA therapy is more reliable than those received a prolonged course of antibiotics), clinical severity, and patient risk factor for MRSA.

Another aspect in MRSA PCR protocol/guideline to deliberate is the necessity of repeating nasal MRSA PCR screening. Two institutions excluded patients who had previous nasal MRSA PCR result or MRSA in respiratory cultures within 7–14 days from their pharmacist nasal MRSA PCR protocols. A study of 736 patients from a community health-system examined NPV of nasal MRSA PCR based on the time between nasal PCR and respiratory samples. NPV of nasal MRSA PCR screen remained high regardless of the time elapsed between nasal MRSA PCR and respiratory culture collection.^18^ The NPVs were 93.8% in ≤ 24 hours group (*n* = 316), 98.6% in 25 to 48 hours group (*n* = 80), 95.7% in 49 hours to 7 days group (*n* = 190), 92.9% in 8–14 days group (*n* = 99), and 95.5% in >14 days group (*n* = 51).^18^ Another study found conversion rate from negative nasal MRSA PCR to positive within 14 days was rare (0.9%).^19^ These data suggest that repeating nasal MRSA PCR within 14 days of a prior test is generally unnecessary, and may serve as an additional factor to consider when developing nasal MRSA PCR screen protocol/guideline.^18,19^

The use of nasal MRSA PCR has become a widely adopted tool in antimicrobial stewardship, and guidelines and protocols are critical factors in its successful integration. Allowing all pharmacists—not just those in stewardship roles—to order nasal MRSA PCR can streamline the process and ensure quicker results. No institutions in Kentucky permit pharmacists to independently discontinue anti-MRSA therapy based on a negative nasal MRSA PCR screen. Future protocols can be designed to enable pharmacists to do so for specific indications, such as uncomplicated pneumonia for which data has consistently demonstrated high NPV. More expansive de-escalation criteria may allow pharmacists to facilitate timely clinical interventions in partnership with treating teams.

## Conclusion

This study represents the first statewide review of nasal MRSA PCR screening practices and reveals variations across multiple institutions in the state of Kentucky. The results may serve as a resource for stewardship programs by providing a range of approaches and allowing institutions to adapt elements that align with their local needs and operational feasibility into their nasal MRSA PCR protocols and guidelines.

## Supporting information

10.1017/ash.2025.10201.sm001 Truong et al. supplementary material Truong et al. supplementary material

## Supplementary material

The supplementary material for this article can be found at https://doi.org/10.1017/ash.2025.10201.
