# Supplementary material for: KY-MRSA: a comprehensive review of methicillin-resistant Staphylococcus aureus polymerase chain reaction nasal screening practices across nine institutions in Kentucky
Source: Antimicrob Steward Healthc Epidemiol. 2025 Oct 30;5(1):e290. doi: 10.1017/ash.2025.10201 (PMC12616569; doi:10.1017/ash.2025.10201)
Supplement: Truong et al. supplementary material [file S2732494X25102015sup001.docx]

**Appendix**

**Protocol 1:**

**SCOPE:** Inpatient Care

**PURPOSE**: The purpose of this policy is to allow the pharmacist to order a methicillin resistant Staphylococcus aureus (MRSA) nares polymerase chain reaction (PCR) as part of a System-wide antimicrobial stewardship initiative to reduce antibiotic use and adverse effects in patients started on therapy.

**DEPARTMENTS AFFECTED**: Pharmacy

**POLICY**: Pharmacists may order an MRSA nares PCR on adult patients within 48 hours of the start of antibiotics when a provider has ordered vancomycin, linezolid, or any other agent for the purposes of treating MRSA. This policy applies to antibiotics started for all antibiotic indications, excluding central nervous system (CNS) infections and surgical prophylaxis.

**PROCEDURE**:

A. When a provider orders an anti-MRSA agent for the purpose of treating MRSA, the pharmacist may order an MRSA nares PCR on adult patients, preferably within 24 hours, but no later than 48 hours beyond the start of anti-MRSA therapy as part of a diagnostic workup to help rule out MRSA as the potential infectious cause, excluding CNS infections.

B. The pharmacist will assess the results and discuss with the team. If the MRSA nares PCR is negative, the pharmacist will then discuss the results, clinical situation, and potential antibiotic de-escalation with the provider

**REFERENCES**:

1. Parente DM, Cunha CB, Mylonakis E, Timbrook TT. The Clinical Utility of Methicillin-Resistant Staphylococcus aureus (MRSA) Nasal Screening to Rule Out MRSA Pneumonia: A Diagnostic Meta -analysis With Antimicrobial Stewardship Implications. Clin Infect Dis. 2018 Jun 18;67(1):1-7.
2. Dangerfield B, Chung A, Webb B, Seville MT. Predictive value of methicillin-resistant Staphylococcus aureus (MRSA) nasal swab PCR assay for MRSA pneumonia. Antimicrob Agents Chemother. 2014;58(2):859 -64.
3. Mergenhagen KA, Starr KE, Wattengel BA, Lesse AJ, Sumon Z, Sellick JA. Determining the Utility of Methicillin -Resistant Staphylococcus aureus Nares Screening in Antimicrobial Stewardship. Clin Infect Dis. 2020 Aug 22;71(5):1142 -1148.
4. Willis C, Allen B, Tucker C, Rottman K, Epps K. Impact of a pharmacist-driven methicillin-resistant Staphylococcus aureus surveillance protocol. Am J Health Syst Pharm. 2017 Nov 1;74(21):1765-1773.
5. Dadzie P, Dietrich T, Ashurst J. Impact of a Pharmacist-driven Methicillin-resistant Staphylococcus aureus Polymerase Chain Reaction Nasal Swab Protocol on the De-escalation of Empiric Vancomycin in Patients with Pneumonia in a Rural Healthcare Setting. Cureus. 2019;11(12):e6378. Published 2019 Dec 13.
6. Baby N, Faust AC, Smith T, Sheperd LA, Knoll L, Goodman EL. Nasal Methicillin-Resistant Staphylococcus aureus (MRSA) PCR Testing Reduces the Duration of MRSA-Targeted Therapy in Patients with Suspected MRSA Pneumonia. Antimicrob Agents Chemother. 2017;61(4):e02432-16. Published 2017 Mar 24.
7. Pham SN, Sturm AC, Jacoby JS, Egwuatu NE, Dumkow LE. Impact of a Pharmacist-Driven MRSA Nasal PCR Protocol on Pneumonia Therapy. Hosp Pharm. 2021 Aug;56(4):221-227.

**Protocol 2**:

**POLICY**: Appropriate cultures of blood (2 sets), sputum, urine, wounds, and normally sterile bodily fluids should be obtained BEFORE the institution of antimicrobial therapy.  If order received for restricted antibiotic without order for appropriate cultures, the clinical pharmacist processing the order may enter an order to obtain the appropriate cultures but should not cause an excessive delay (>1 hour to initiation of antimicrobial agent), (i.e. blood cultures should be ordered “stat”).  Other diagnostic tests may be ordered by a pharmacist to assist with opportunities for antimicrobial de-escalation (e.g. procalcitonin, urine antigens, etc.).

**PROCEDURE**: Pharmacists will order MRSA nasal swab when vancomycin or linezolid is ordered with clinical indication of pneumonia.

**Protocol 3:**

**POLICY:** Pharmacists may order an MRSA PCR swabs on patients when provider consult pharmacy for vancomycin or linezolid for pneumonia.

**Protocol 4:**

**PURPOSE**: To outline the responsibilities of clinical pharmacists when consulted to dose vancomycin for pneumonia.

**POLICY**:

**Responsible Persons**: All Clinical Pharmacy Staff

**GUIDELINES:**

1. Providers are required to enter indications for antimicrobials per hospital policy.
2. When consulted to dose vancomycin specifically for pneumonia in adult patients only, clinical pharmacists should follow the outlined procedures
3. Exclusions:
   1. Patients with an existing nasal MRSA PCR swab within the last 7 days
   2. Confirmed MRSA in respiratory or nasal culture within the last 14 days

**PROCEDURE:**

1. When consulted to dose vancomycin for pneumonia, pharmacists should order a nasal MRSA PCR swab. Orders should be entered under the consulting provider and signed as "Per Pharmacy Consult (No Cosign Required)."
   1. See Guidelines, Sec. 3 for exclusions
2. Negative results should be relayed to attending provider by a clinical pharmacist within 24 hours of final result.

**REFERENCES:**

1. Lance R. Peterson, Molecular Laboratory Tests for the Diagnosis of Respiratory Tract Infection Due to Staphylococcus aureus, Clinical Infectious Diseases, Volume 52, Issue suppl_4, May 2011, Pages S361–S366.
2. Parente DM, Cunha CB, Mylonakis E, Timbrook TT. The Clinical Utility of Methicillin-Resistant Staphylococcus aureus (MRSA) Nasal Screening to Rule Out MRSA Pneumonia: A Diagnostic Meta-analysis With Antimicrobial Stewardship Implications. Clin Infect Dis. 2018 Jun 18;67(1):1-7.
3. Phar S, Sturm AC, Jacoby JS, Eygue AL. The Impact of a Pharmacist-Driven MRSA Nasal PCR Protocol on Pneumonia Therapy. Hosp Pharm. 2021 Aug;56(4):221-227.
4. Baby N, Faust AC, Smith T, Sheperd LA, Knoll L, Goodman EL. Nasal Methicillin-Resistant Staphylococcus aureus (MRSA) PCR Testing Reduces the Duration of Anti-MRSA Targeted Therapy in Patients with Suspected MRSA Pneumonia. J Antimicrob Chemother. 2017 Mar 1;45(1):e0236216.
5. Meng L, Protti A, Hitchcock MM, Hall MB, Diep AL, Wargo JJ, Eto CD, Swaisgory R, Chang R, Barnett N, Dresser SJ, Holubar M. Discontinuation Patterns and Cost Avoidance of a Pharmacist-Driven Methicillin-Resistant Staphylococcus aureus Nasal Polymerase Chain Reaction Testing Protocol for De-escalation of Empiric Vancomycin for Suspected Pneumonia. Open Forum Infect Dis. 2021 Mar;8(4):ofab089.

**Protocol 5:**

The full protocol was not shared due to internal policy restrictions

**Guideline 1a (Pediatrics):**

**Guideline for the Management of Community-Acquired Pneumonia in Pediatrics**


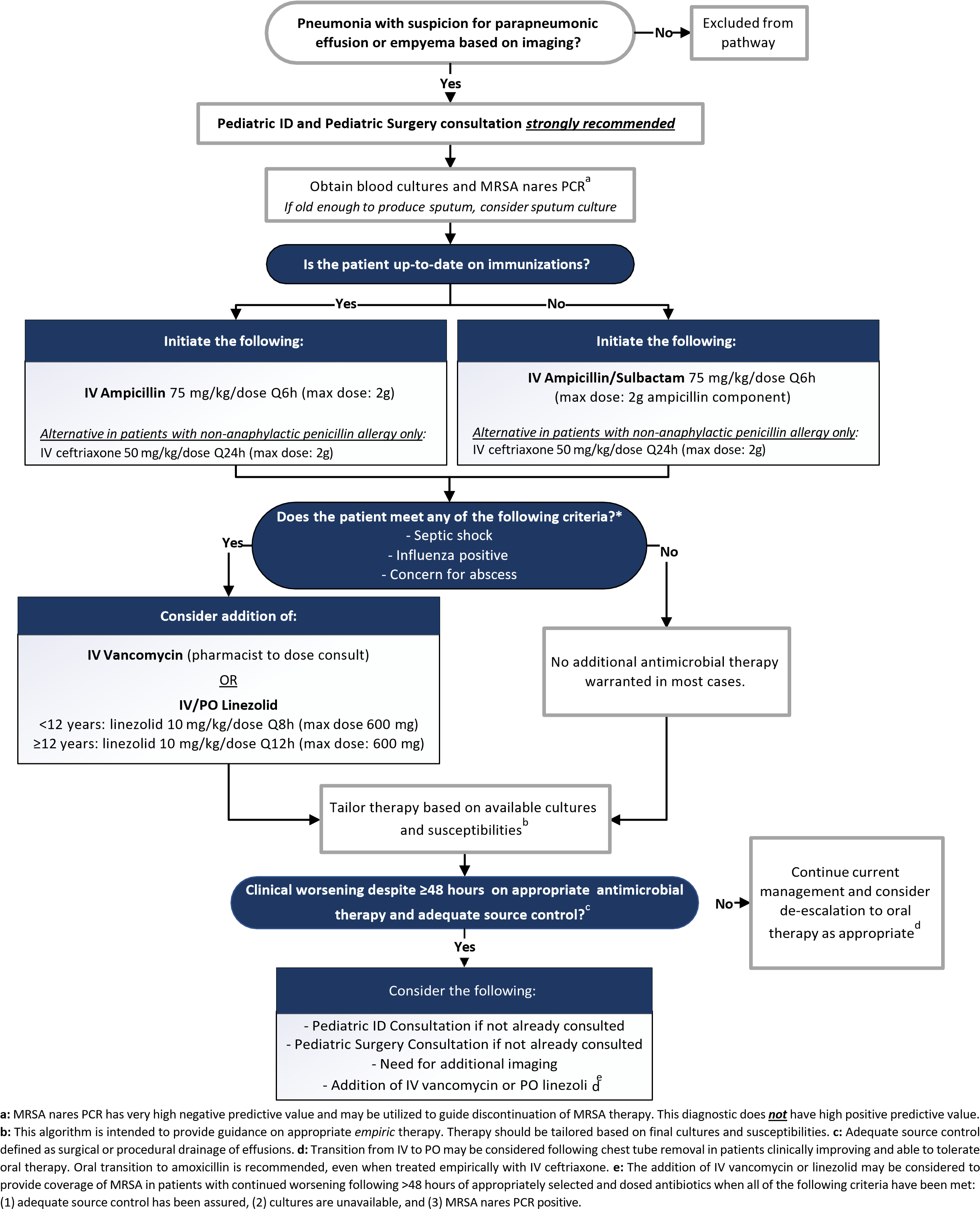


**Management of Pediatric Patients with Suspected Preseptal or Orbital Cellulitis**


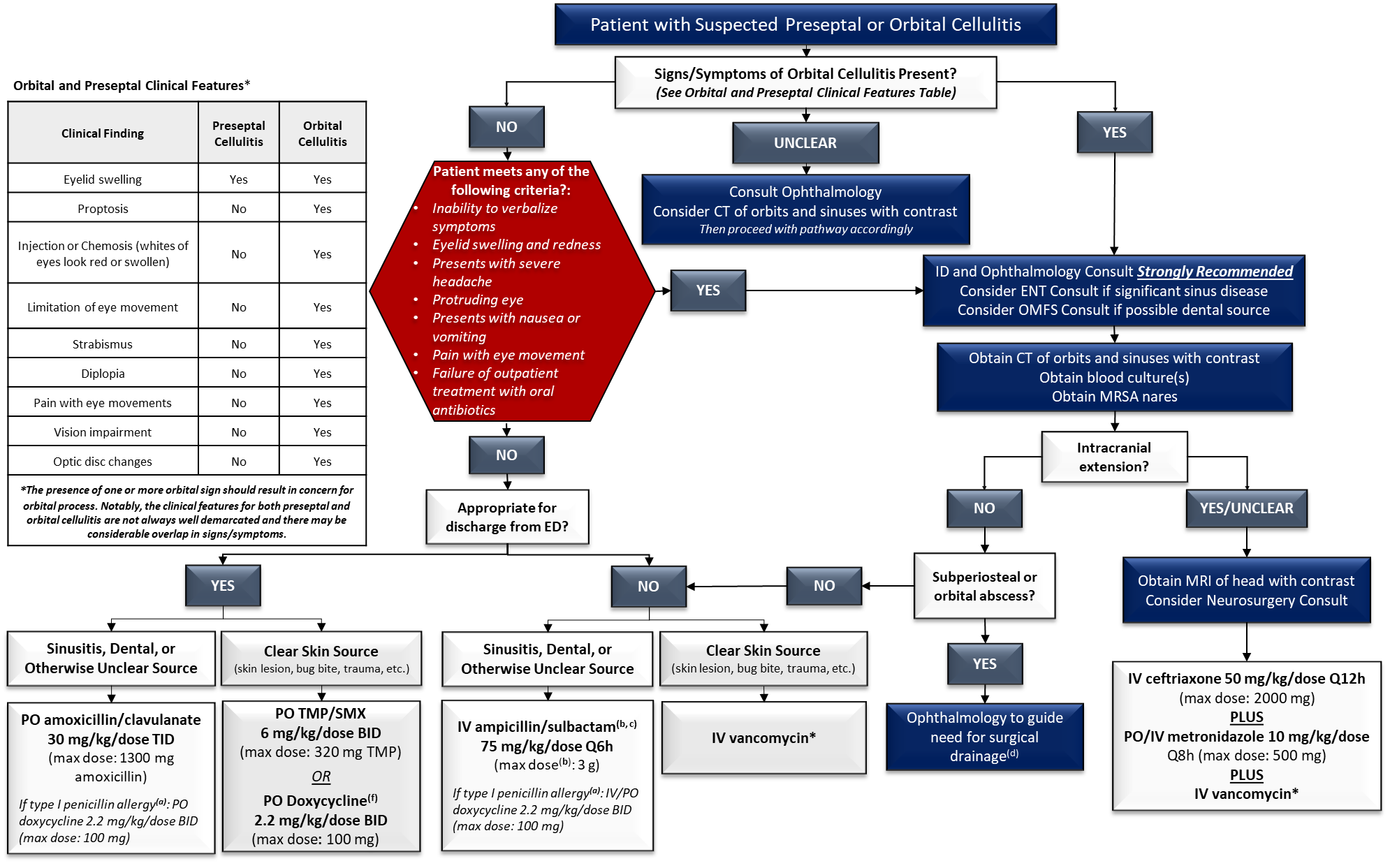


**Guideline 1b (Adults):**

**Nosocomial Pneumonia Guideline**

**MRSA Nasal Screening**

- - MRSA PCR-based nares screening should be utilized in patients with HAP and VAP to assist in the de-escalation of MRSA targeted antibiotics (for example, linezolid or vancomycin).
- Background: MRSA is a common colonizer of the nares. In a 2018 meta-analysis, the negative predictive value (NPV) for MRSA pneumonias was found to be 96.5%. Clinical outcomes, including mortality rates, have been noted to be similar among patients with therapy de-escalations derived from MRSA nasal screens. The evidence is strongest in patients with HAP, where the evidence in VAP remains less clear as MRSA can be introduced by an alternative route (artificial airway). Screening is still recommended in VAP as NPV has been reported to be 94.8%; however, it is important to take an individualized approach when considering de-escalating in this population (consider hemodynamic stability, history of MRSA infection etc.).
- Considerations
  - - - PCR-based screening is preferred. Culture-based screens are performed in the ICUs on a weekly basis and may be used if available.
      - MRSA screen results can be utilized if known within a week prior to pneumonia diagnosis.
      - MRSA nares screens do not have high positive predictive value in the diagnosis of MRSA pneumonia and should only be used as a marker for de-escalation.
        - If MRSA nares PCR is positive, follow respiratory culture for de-escalation
      - Screening should not be used for therapy decisions in patients with recent (within 30 days) nasal MRSA decolonization. This occurs in pediatric and adult patients with a foley catheter, central line, or history of MRSA, as well as all ICU patients.
      - Nasal screening *may* occur after initiation of therapy as parenteral antimicrobials are ineffective at clearing nasal colonization and MRSA persists in the respiratory tracts for the first few days of therapy.
